# Ventilator-associated pneumonia in critically ill patients with COVID-19

**DOI:** 10.1186/s13054-021-03460-5

**Published:** 2021-01-11

**Authors:** Mailis Maes, Ellen Higginson, Joana Pereira-Dias, Martin D. Curran, Surendra Parmar, Fahad Khokhar, Delphine Cuchet-Lourenço, Janine Lux, Sapna Sharma-Hajela, Benjamin Ravenhill, Islam Hamed, Laura Heales, Razeen Mahroof, Amelia Solderholm, Sally Forrest, Sushmita Sridhar, Nicholas M. Brown, Stephen Baker, Vilas Navapurkar, Gordon Dougan, Josefin Bartholdson Scott, Andrew Conway Morris

**Affiliations:** 1 Department of Medicine, Cambridge Institute of Therapeutic Immunology and Infectious Disease (CITIID), University of Cambridge, Cambridge, UK; 2 Public Health England, Clinical Microbiology and Public Health Laboratory, Addenbrooke’s Hospital, Cambridge, UK; 3 Division of Anaesthesia, Department of Medicine, University of Cambridge, Level 4, Addenbrooke’s Hospital, Hills Road, Cambridge, CB2 0QQ UK; 4 John Farman ICU, Addenbrookes Hospital, Cambridge, UK; 5 Wellcome Sanger Institute, Hinxton, UK

**Keywords:** COVID-19, SARS-CoV-2, Nosocomial infections, Molecular diagnostics, Ventilator-associated pneumonia, Critical care

## Abstract

**Background:**

Pandemic COVID-19 caused by the coronavirus SARS-CoV-2 has a high incidence of patients with severe acute respiratory syndrome (SARS). Many of these patients require admission to an intensive care unit (ICU) for invasive ventilation and are at significant risk of developing a secondary, ventilator-associated pneumonia (VAP).

**Objectives:**

To study the incidence of VAP and bacterial lung microbiome composition of ventilated COVID-19 and non-COVID-19 patients.

**Methods:**

In this retrospective observational study, we compared the incidence of VAP and secondary infections using a combination of microbial culture and a TaqMan multi-pathogen array. In addition, we determined the lung microbiome composition using 16S RNA analysis in a subset of samples. The study involved 81 COVID-19 and 144 non-COVID-19 patients receiving invasive ventilation in a single University teaching hospital between March 15th 2020 and August 30th 2020.

**Results:**

COVID-19 patients were significantly more likely to develop VAP than patients without COVID (Cox proportional hazard ratio 2.01 95% CI 1.14–3.54, *p* = 0.0015) with an incidence density of 28/1000 ventilator days versus 13/1000 for patients without COVID (*p* = 0.009). Although the distribution of organisms causing VAP was similar between the two groups, and the pulmonary microbiome was similar, we identified 3 cases of invasive aspergillosis amongst the patients with COVID-19 but none in the non-COVID-19 cohort. *Herpesvirade* activation was also numerically more frequent amongst patients with COVID-19.

**Conclusion:**

COVID-19 is associated with an increased risk of VAP, which is not fully explained by the prolonged duration of ventilation. The pulmonary dysbiosis caused by COVID-19, and the causative organisms of secondary pneumonia observed are similar to that seen in critically ill patients ventilated for other reasons.

## Background

Pandemic COVID-19 is associated with a high number of patients suffering from severe acute respiratory syndrome (SARS). Such patients can spend significant periods of time in intensive care units (ICU), with up to 80% of patients admitted to ICU requiring invasive mechanical ventilation [1, 2]. Critically ill patients are at high risk of nosocomial pneumonia, especially when ventilated [3]. The reasons for this includes breach of natural defences by invasive devices [4], sedation and impairment of coughing and mucociliary clearance, and the immunoparetic effects of critical illness [5, 6]. Early reports indicated that critically ill patients infected with SARS-CoV-2 had a high prevalence of nosocomial pneumonia, especially ventilator-associated pneumonia (VAP) [7]. More recent reports, including a large survey from a single hospital [8] and a synthesis of the literature [9] suggested that rates of secondary infections were low, although neither study focussed specifically on critical care. There remains considerable uncertainty around the incidence of nosocomial infections in severe COVID-19, which has led to recent calls for more analysis on the frequency, timing, and causative organisms of these important adverse events [10].

Reports of ICU-acquired infection in patients with COVID-19 have been limited and have often not reported the details of the causative organisms [7], or have focused on the incidence of one particular infection such as invasive aspergillosis [11]. Importantly, we are not aware of reports of ICU-acquired infections comparing patients with COVID-19 and those without managed contemporaneously within the same settings, which is key to interpreting the frequency, timing, and causative organisms leading to these infections.

Ventilator-associated pneumonia (VAP), the commonest ICU-acquired infection [3], can be challenging to diagnose as a range of non-infectious diseases may mimic the clinical picture of radiographic infiltrates, systemic inflammation and impaired oxygenation that typifies VAP [12]. To limit overdiagnosis and facilitate appropriate antimicrobial therapy in VAP, guidelines advocate the use of culture- based approaches [13, 14]. However, molecular tests to detect multiple pathogens (viruses and bacteria) are becoming more accessible and may further reduce unnecessary antimicrobial therapy [15] whilst enhancing the detection of hard to culture organisms.

During our hospital’s first wave of COVID-19 admissions we noted an apparent increase in the rate of VAP. In this study, we therefore aimed to identify and compare the distribution of VAP in critically ill ventilated COVID-19 patients compared to ventilated non-SARS-CoV-2 infected patients admitted to the same unit. We performed conventional microbiological culture on all lower respiratory tract samples. Bronchoalveolar lavage (BAL) was also analysed using a multi-pathogen TaqMan array card (TAC) we have developed and reported previously [16]. In a sub-set of BALs we assessed the composition of the bacterial lung microbiome in bronchoalveolar lavage (BAL) by 16S sequencing.

## Materials and methods

### Setting and study design

**Fig. 1:**
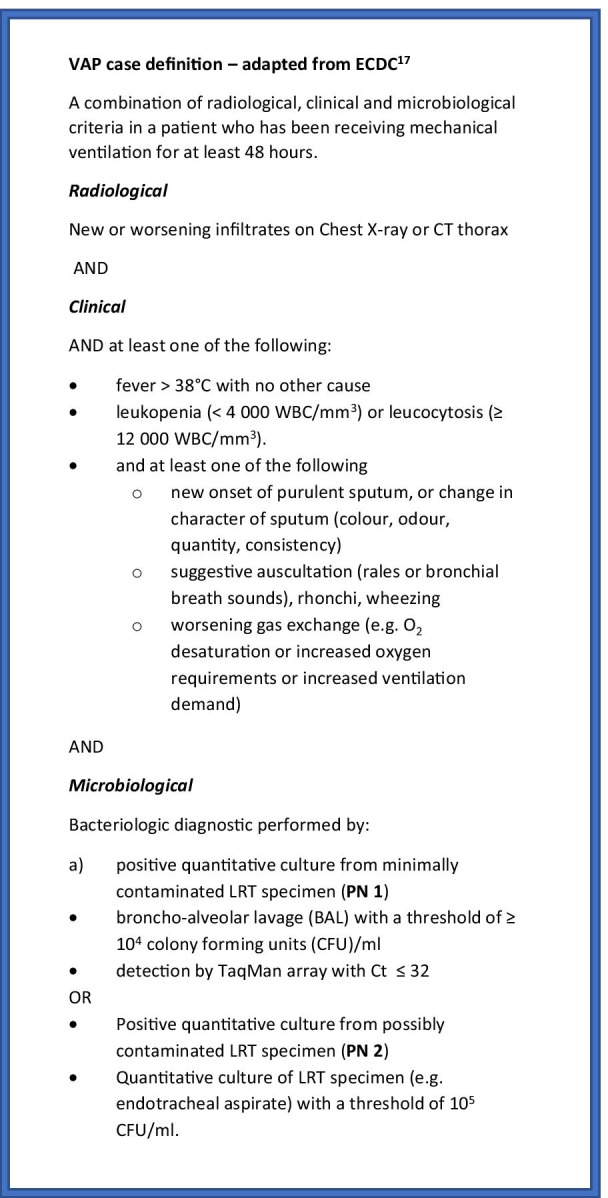
Criteria used for the diagnosis of VAP. Adapted from the European Centre for Disease Control definitions to meet local thresholds for quantitative culture of endotracheal aspirate and for the inclusion of molecular detection of pathogens. Ct-cycles to threshold by quantitative PCR

This study was performed in the liver/general adult ICU in Addenbrooke’s Hospital, Cambridge, UK, and also included COVID-19 patients managed in the neurotrauma and dedicated COVID-19 ICUs of the hospital. Patients were reviewed at least twice daily by consultant intensive care physicians with investigation for VAP ordered by this clinician, and discussed at a daily microbiology-intensive care multi-disciplinary team/antimicrobial stewardship meeting. We had a regularly audited ventilator bundle in place, which consisted of sub-glottic suction endotracheal tubes, mandated twice daily oral hygiene with fluoride toothpaste, daily sedation holds and head of bed elevation. One to one nursing to patient ratios were maintained throughout the first wave of COVID-19, although at times this included nurses with limited critical care training as normal ICU capacity was exceded. Sessional use of personal protective equipment (full-length fluid impermeable gowns, FFP3 mask, gloves and hat) with apron and second glove change between patients was maintained from March 15th to July 31st. Patients ventilated for at least 48 h, from March 15th (date of our first COVID-19 admission) to August 30th were retrospectively reviewed for presence of VAP. VAP was defined using a modification of the European Centre for Disease Control definitions [17] for quantitative BAL culture (termed PN1) or quantitative endotracheal aspirate (ETA) or sputum culture (termed PN2) definitions of pneumonia (see Fig. 1). The modifications were to use polymerase chain reaction (PCR) positivity by TAC for BAL fluid (details below) and to use a threshold of ≥ 10^5^ Colony Forming Units (CFU)/ml for endotracheal aspirate in keeping with UK standards [18]. Low lung pathogenicity organisms (*Enterococcus* spp., *Candida albicans*, non-pneumococcal *Streptococci* and coagulase negative *Staphylococci*) were reported but not considered a component of VAP [19]. *Herpesviridae* (Herpes simplex, cytomegalovirus and Epstein-Barr virus) were reported but were considered to be reactivations and not considered a component of VAP [20].

We also looked for evidence of invasive pulmonary aspergillosis (IPA), as there are now several case reports of this developing in patients with COVID-19 [11] and recent reports of its frequency in non-COVID VAP [21]. IPA was defined using the criteria set out in the report describing influenza associated pulmonary aspergillosis [22] modified to include diagnosis by PCR. The criteria were clinical evidence of pulmonary infection, radiological evidence of pulmonary infection and detection of aspergillus by BAL galactomannan, PCR positivity or culture positivity.

### Diagnostics

Samples for routine microbiology were processed according to the UK Standards for Microbiology Investigations [18]. Any significant growth with a CFU of ≥ 10^4^/mL (on BAL) or ≥ 10^5^/mL ETA was identified by MALDI-ToF mass spectrometry. Our lab also routinely runs a multipathogen TaqMan array on bronchoalveolar lavage samples [16], the details of this are noted below.

### TaqMan multi-pathogen array

Custom designed TaqMan Array Cards (TAC; Thermo Fisher Scientific) targeting 52 different common respiratory pathogens, were used to test for secondary infections as previously described [16]. Detection of a clear exponential amplification curve with a Cycles to Threshold (CT) value ≤ 32 for any single gene target was reported as a positive result for the relevant pathogen. We have previously demonstrated that CT value of ≤ 32 corresponded to growth ≥ 10^4^/CFU/ml, hence the use of this threshold to define VAP [16]. Details of the procedures for extraction of nucleic acids for TAC, SARS-CoV-2 qPCR and 16S DNA nanopore sequencing are contained in the supplemental methods.

### Statistical analysis

The primary analysis was time to development of first VAP, censored for extubation or death, with comparison by univariable Cox proportional hazards model. Secondary analysis of VAP as an incidence density (cases per 1000 ventilator days) compared with Mid-P exact test.

Risk factors for VAP were compared using a Cox proportional hazards model, with variables rejected if their p value was > 0.05 on univariable analysis, statistically significant variables entered the final model. Analyses were conducted using SPSS (v25 IBM, Armonk, NY).

## Results

Overall, we managed 94 patients with COVID-19, of whom 81 were ventilated for more than 48 h. From the period 15th March to 30th August we also managed 144 patients without COVID-19 in the liver/general unit who required ventilation for more than 48 h. The demographic and clinical features of these two groups are shown in Table 1 and details of non-COVID admission diagnoses in Additional file 1: Table S1. Ventilator bundle audit data demonstrated high compliance (compliance with the full bundle ranged from 85 to 100%, with 99–100% for the period April–May when most COVID-19 patients were admitted).

**Table 1:** Clinical and demographic features of reported populations

Parameter	COVID-19 (*n* = 81)	Non-COVID-19 (*n* = 144)	*P* value
Median age (IQR)	62 (50–70)	62 (49–72)	0.986
Sex (*n* (%) female)	25 (31%)	58 (40%)	0.254
Hypertension	27 (33%)	47 (33%)	0.96
Diabetes	18 (22%)	34 (24%)	0.72
Obesity	30 (37%)	34 (24%)	0.04
Chronic kidney disease	10 (12%)	13 (9%)	0.47
Chronic lung disease	16 (20%)	34 (24%)	0.38
Immunocompromised*	12 (15%)	36 (25%)	0.08
Corticosteroid use in ICU	13 (16%)	23 (16%)	0.99
Median APACHE II (IQR)	15 (11–19)	16 (12–20)	0.06
% With ARDS on ICU admission	63 (78%)	21 (15%)	< 0.0001
% Ventilated prone	40 (49%)	1 (0.7%)	< 0.0001
Median P/F ratio in 24 h following admission	18 (13–28)	34 (24–37)	< 0.0001
Antibiotics in 24 h following admission	76 (94%)	126 (88%)	0.23
Median ICU length of stay (IQR)	15 (11–25)	9 (4–13)	< 0.0001
Median duration of ventilation (IQR)	14 (10–23)	5 (2–11)	< 0.0001
% Developing suspected VAP	64 (79%)	48 (33%)	< 0.0001
% Developing microbiologically confirmed VAP	39 (48%)	19 (13%)	< 0.0001
% of suspected VAPs investigated by bronchoscopy and lavage	30 (47%^#^)	23 (48%^##^)	0.94
ICU mortality	31 (38%)	30 (21%)	0.006
Median length of stay for patients dying in ICU (IQR)	13 (10–17)	9 (6–11)	0.0019

*P* value by *z*-test for proportions and by Mann–Whitney *U* test for continuous variables

^*^Immunocompromised patients were defined as having active haematological malignancy, neutropaenic malignancy, solid organ or bone marrow transplant and receipt of immunosuppressive medication including corticosteroids for > 1 week prior to hospital admission

^#^4 cases not assessed by TAC due to lack of availability of laboratory capacity

^##^1 case not assessed by TAC due to lack of availability of laboratory capacity

APACHE II, Acute physiology and chronic health evaluation II score; IQR, interquartile range

Patients with COVID-19 were significantly more likely to be investigated for VAP (Table 1), and had a higher incidence of microbiologically confirmed VAP (39 (48%) COVID-19 patients compared to 19 (13%) patients without COVID-19). Further details of the comparison of the investigation for VAP are shown in Additional file 1: Tables S2 and S3. Patients who were investigated for VAP demonstrated a significant deterioration in oxygenation relative to the period immediately prior to the diagnosis (Additional file 1: Figure S1).

**Fig. 2:**
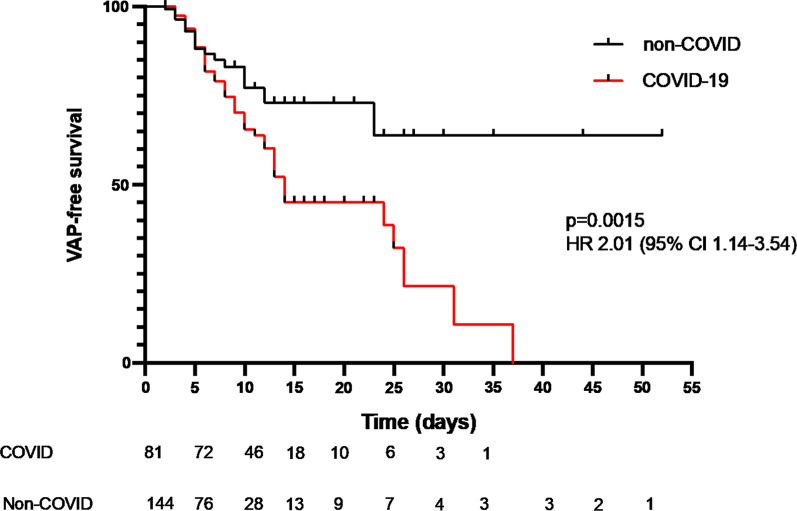
Time to development of VAP in patients with and without COVID-19 censored for death or extubation. P value and hazard ratio by Cox proportional hazards. Numbers at risk at each time point indicated below x-axis

Survival analysis (Fig. 2) demonstrated that the increased risk of developing VAP in patients with COVID-19 was not simply a function of longer duration of ventilation. The hazard of early VAP was similar in both groups of patients, however the greater number of later VAPs in COVID-19 led to the increased median duration of ventilation before VAP developed seen in Additional file 1: Table S2. The effect of COVID status on VAP-free survival remained significant when adjusted for age and immunocompromised status (adjusted p value 0.045 by Cox proportional hazards model, Additional file 1: Table S5). Sensitivity analysis of patients with > 72 h mechanical ventilation and > 144 h of mechanical ventilation produced similar survival curves and hazard ratios (Additional file 1: Figure S2A and B). A similar finding was apparent when comparing crude incident density, patients with COVID-19 developed VAP at a rate of 28/1000 ventilator days, whilst those without COVID-19 experienced VAP at a rate of 13/1000 ventilator days (*p* = 0.009 by mid-P exact test). Incident density censoring for post-VAP duration ventilation, which is confounded by VAP itself prolonging ventilation, shows a similar pattern (40/1000 ventilator days for COVID-19, 19/1000 ventilator days for non-COVID *p* = 0.004 by mid-P exact test). Further details on timing of VAP are available in the supplemental section (Additional file 1: Table S2). Antibiotic use on admission (Table 1) and in the period leading up to investigation for suspected VAP (Additional file 1: Tables S3 and S4) was similar in frequency and spectrum of agents used.

The organisms identified on endotracheal aspirate culture and both culture and molecular testing of bronchoalveolar lavage fluid are show in Table 2. The concordance between culture and molecular testing was high, although molecular testing identified a number of additional organisms.

**Table 2:** Organisms identified by culture and PCR testing (BAL only) in patients with confirmed VAP

Organism	ETA (≥ 10^5^ CFU/ml)	BAL culture (≥ 10^4^ CFU/ml)	BAL PCR (≤ Ct32)
**Gram negative**			
*Burkholderia cepacia*	1		
*Citrobacter freundii*		1	*
*Citrobacter koseri*	1	1	*
Coliform (not further specified)	1		
*Escherichia coli*	5	3^!^	4^!^
*Enterobacter asburiae*	1		*
*Enterobacter cloacae*	3		
Enterobacteraeciae (not further specified)			2
*Haemophilus influenzae*	1		4
*Klebsiella aerogenes*	2	1	*
*Klebsiella pneumoniae*	2	3	5
*Klebsiella oxytoca*	3	1	*
*Proteus mirabilus*	1		1^$^
*Pseudomonas aeruginosa*	7	3	2
*Serratia liquefaciens*	1		
*Serratia marcescens*	1	2	5
*Stenotrophomonas maltophilia*	3	4	4
**Gram positive**			
*Staphylococcus aureus*	2	2	1
**Fungi**			
*Aspergillus fumigatus*			1
**Non-pathogenic organisms**			
*Candida albicans*	6	4	4
*Candida* spp.			1
Coagulase negative *Staphylococci*		1	4
*Enterococcus faecium*		2	7
*Streptococcus* spp. (non-pneumoniae, non-pyogenes)			4
Herpes simplex virus			2

^*^Sequence for the organism in question not present on the TAC

^!^1 *E. coli* was detected by culture but not TAC in a patient, 2 *E. coli* detected by TAC without growth on culture

^$^Sequence on TAC is for *Proteus* spp. rather than species specific

**Fig. 3:**
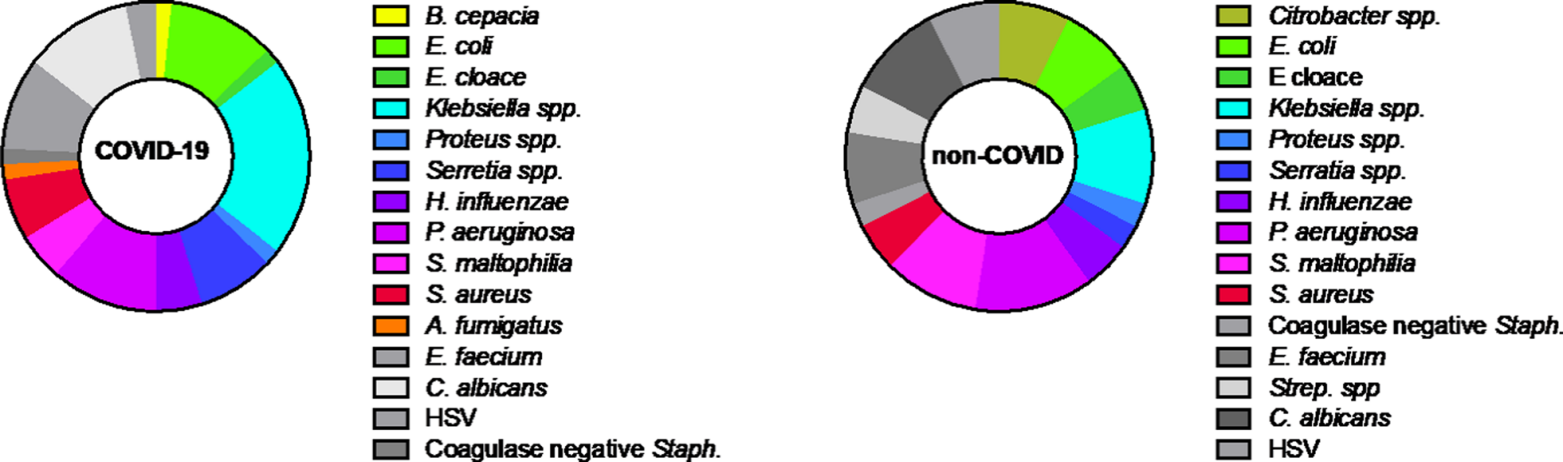
Causative organisms of VAP in patients with and without COVID-19. Non-pathogenic organisms detected above threshold levels shown in grey

The distribution of organisms in COVID-19 and non-COVID-19 associated VAP is shown in Fig. 3, and is broadly similar between both groups.

### Lung microbiota

**Fig. 4:**
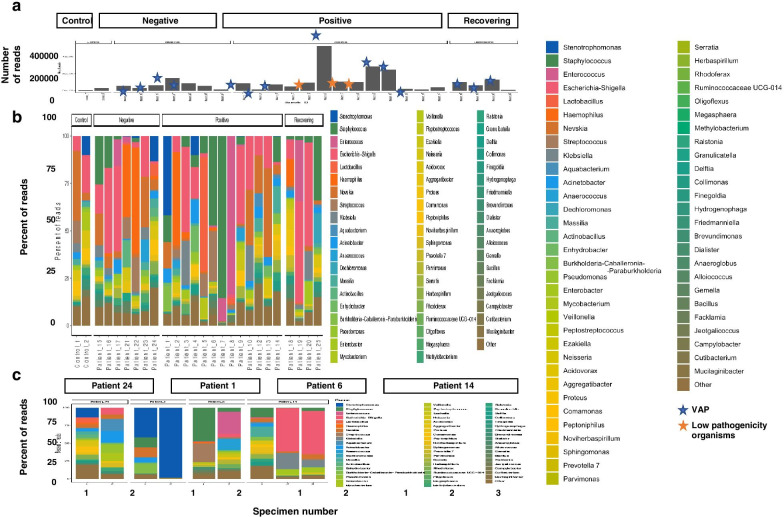
Microbial composition of BAL samples from SARS-CoV-2 positive and negative patients. Bacterial 16S genes were sequenced and classified to the genus level using Kraken2. The number and percent of reads mapping to each genus is shown for individual samples from each patient (**A**), with kit controls in the first two columns, and longitudinal samples (1, 2 or 3) from individual patients (**B**). Individuals were classified as either COVID-19 negative, COVID-19 positive, or recovering (previously diagnosed with COVID-19 but SARS-CoV-2 negative at time of sample)

To investigate changes in the lung microbiota in the COVID-19 positive and negative patients we performed 16S rRNA sequencing on a subset of BAL samples from 24 patients. In general, bacteria detected by TAC or conventional microbiology were abundantly identified in samples by 16S sequencing (Fig. 4). Samples with confirmed VAP or colonization with low pathogenic organisms generally yielded higher overall read numbers. When comparing COVID-19 positive to COVID-19 negative patients, there was no specific taxon that was more prevalent in either group. Additionally, in this relatively small subset of samples, the bacterial composition of BALs from COVID-19 positive patients were not significantly different in either the species richness (alpha diversity) or the microbial composition (beta diversity).

To investigate changes in the microbiota over the course of infection, we next looked at the microbial composition of BAL samples in some individual patients over time. Two patients diagnosed with VAP (patients 1 and 24) showed decreasing species richness over time, as the bacterial pathogen implicated in the illness became the predominant microbe present. For patient 6, the microbial composition shifted significantly over time, as *Enterococcus* took over from *Staphylococcus* as the most predominant organism. The microbiome composition of patient 24, who was both VAP and COVID-19 negative, was largely stable over time. In general, the microbial composition of BAL samples from patients who did not have VAP at the time of sampling (sample 1 from patient 14 and both samples from patient 24) were more diverse than samples from patients who had been diagnosed with VAP.

### Invasive aspergillosis

43 patients were investigated for possible pulmonary aspergillosis by PCR and lavage galactomannan, on the basis of senior clinician suspicion of fungal infection. 23 patients with COVID-19 and 20 without. Of these 3 COVID-19 patients met the criteria for IPA outlined in the methods above (one positive by PCR with borderline galactomannan 0.7 optical density index (ODI), and 2 PCR negative but with galactomannan > 1.0 ODI), and all were treated with liposomal amphotericin, 2 of these patients survived to hospital discharge whilst one died. One patient without COVID-19 had a borderline positive galactomannan (0.8 ODI), and met clinical criteria but was not treated as care was withdrawn for other reasons. We estimate the prevalence of COVID-19-associated aspergillosis (CAPA) to be 13%, although with small numbers the confidence intervals are wide (95% CI 5–32%). None of the three patients with CAPA had received steroids prior to the diagnosis.

### Reactivation of herpesvirade

49 patients had lavage tested for herpesvirade, 24 with COVID-19 and 25 without. Although five patients (two with VAP from other organisms, and three without VAP) had detection of herpes simplex virus (HSV) below the Ct cut-off of 32, in viral reactivation the role of viral load is uncertain. We therefore examined the frequency of herpesvirade detection at any level in lavage of patients investigated for suspected VAP. In total 10 patients with COVID-19 had detection of herpesvirade (4 HSV, 5 Epstein barr virus (EBV) and 1 patient with both), whilst 5 patients without COVID-19 had detection (2 HSV, 1 cytomegalovirus, 1 EBV and 1 patient with both HSV and EBV). As only lavage was tested for herpesvirade, the prevalence of herpesvirade detection amongst the tested population was 42% (95% CI 24–61%) in patients with COVID-19 and 20% (95% CI 9–39%) in patients without COVID-19 (distribution of Ct values for herpesvirade are shown in Additional file 1: Figure S3). Only one patient with herpesvirade activation had received steroids prior to detection.

## Discussion

COVID-19 is a new disease in the human population and this has led to an increase in the number of patients in need of mechanical ventilation, which in turn introduces the risk of VAP. COVID-19 can present in various severe manifestations and reports of co-infections vary [7–9]. However, often these reports suffer from a lack of clarity around the severity of illness, location of patients (critical care vs non-critical care), timing of sampling relative to onset of disease and, where applicable, the use of mechanical ventilation. Here, we report on the most severely affected COVID-19 patients who required ICU admission with mechanical ventilation. We found that relative to patients without COVID-19, the hazard of VAP was significantly elevated.

Conventional surveillance for VAP uses incidence density, which we calculated to allow comparison with previously published reports. We found a high incidence density of confirmed VAP (28/1000 ventilator days) amongst patients with COVID-19, whilst those without COVID-19 had rates closer to those reported from other units in the pre-COVID-19 era, where incident densities were 6–14/1000 ventilator days for confirmed VAP were reported [23]. As sessional use of personal protective equipment remained in place until the end of July 2020, for management of both COVID-19 and non-COVID-19 patients, we do not think that this influenced the differential acquisition of VAP amongst these two groups.

The distribution of infecting organism was similar between patients with and without COVID-19, and reflects that reported in the literature from previous surveys of ICU-acquired infections from before the COVID era [3, 19]. The use of the TAC allowed more rapid identification of organisms, most of which were subsequently identified by culture. Notably there were a few organisms the TAC did not detect, largely because sequences for these organisms were not present on the card, these were distributed between both the COVID-19 and non-COVID patients.

At the lung microbiome level, we observed no difference in the composition of organisms between COVID-19 positive and non-COVID patients who developed VAP. Reassuringly, antibiotic susceptibility of the causative pathogens was similar in the two groups (data not shown) and this meant that conventional antimicrobial regimens could be used.

There is increasing recognition of fungal infections amongst patients with viral pneumonitides and VAP [11, 21, 22]. Although debate continues regarding the differences and similarities between influenza and COVID-associated aspergillosis [10], in keeping with our findings in bacterial VAP it appears that IPA is more common in COVID-19 patients than in ICU patients without COVID-19. It has been suggested that CAPA may relate to the use of immunosuppressive medications [10]. As can be seen from Table 1, steroids were relatively rarely used in this cohort of COVID-19 patients who were largely admitted before the results of the RECOVERY trial had been announced [24] and indeed none of the 3 CAPA patients we identified had received steroids prior to their diagnosis or had underlying immunosuppressive conditions.

More broadly, in our setting immunomodulatory medications were not commonly used at the time of the peak of the COVID-19 admissions, yet there remains a high prevalence of bacterial VAP in these patients. Although VAP in COVID-19 may present problems of quantity, we did not find evidence in this report of a qualitative difference in terms of the organisms causing infection, although as noted above aspergillosis may be more common although this needs to be seen in the context of a significantly higher rate of VAP overall. In the subset where we undertook microbiome profiling, our patients demonstrated similar profiles to those reported by other groups investigating the pulmonary microbiome of ventilated patients [25, 26]. The factors which lead to pulmonary dysbiosis in critical illness remain incompletely understood, but may include intercurrent antibiotic use, enteric translocation, pulmonary immune dysfunction and altered clearance [27].

Although patients without COVID-19 developed proportionately more ‘early’ VAP, being VAP within the first 4 days of ventilation (Additional file 1: Table S2), examination of the VAP-free survival curves (Fig. 2) reveals the hazard of early VAP is similar between the two groups. What is striking, however, is the ongoing risk of VAP seen in patients with COVID-19 which is greater than that seen in patients without COVID. This ongoing risk is reminiscent of the effect we reported previously in critically ill patients with marked immunoparesis [5].

Although from our observational study we cannot be certain why ventilated patients with COVID-19 have such a significantly increased risk of infection, previous work has indicated that the strongest predictor of nosocomial infection in critically ill patients is impaired immune cell function [5, 28]. Patients with COVID-19, in keeping with other critical illness syndromes such as bacterial sepsis and major trauma, experience a complex dysregulation of their immune function with features of both hyperinflammatory activation and organ damage as well as impaired antimicrobial functions [6, 29]. Notably, one of the key drivers of neutrophil impairment in critical illness is the complement component C5a [30, 31] and high levels of complement activation and C5a release have been reported in COVID-19 [32]. Other recent reports on the immunology of COVID-19 highlight marked increases in markers of immune cell functional suppression in the most severely unwell patients [29]. Damage to the alveolar membrane, although not specific to COVID-19, may also facilitate invasion of bacterial species [33]. The estimates of the prevalence of invasive pulmonary aspergillosis and herpesvirade reactivation are limited to those patients investigated by broncho-alveolar lavage and therefore represent only a subset of those investigated for VAP. In the case of invasive aspergillosis it also required senior clinician ordering of galactomannan, and as a retrospective study we cannot be sure clinicians had a common threshold for requesting this test. We therefore acknowledge that these data may underestimate the prevalence of these conditions, however the trend towards higher prevalence amongst patients with COVID-19 adds some support to the hypothesis that these patients suffer from a considerable burden of immunoparesis. It is notable, if not surprising, that patients with COVID-19 were much more likely to present with acute respiratory distress syndrome (ARDS) (Table 1) and consequently had more severe oxygenation defects and were much more likely to be ventilated prone. ARDS is an established risk factor for VAP [34], and the intense pulmonary inflammation can lead to immunologic reprogramming which can impair anti-microbial responses [35]. Prone positioning may increase risk of microaspiration, however dissecting out the specific effects of proning as opposed to the severity of the underlying lung inflammation remains challenging [36]. Whilst previous broad-spectrum antimicrobial therapy is an acknowledged risk factor for VAP [15] we did not find evidence of substantial differences in either antibmicrobial use or spectrum in patients with and without COVID19.

VAP remains difficult to definitively confirm without histological confirmation, which is seldom practical nor desirable in ventilated patients, we therefore cannot be certain that the patients with positive microbiology had definite pneumonia, although the use of quantitative cultures reduces the risk of detection of colonisation as opposed to infection [17, 37]. We used a clinically relevant definition similar to that used in previous studies [17, 37] and applied this consistently across the two groups. We note that diagnostic technique can alter the rate of diagnosis [37], and therefore think it is reassuring that the proportion of bronchoscopic diagnoses was consistent across both groups (Table 1). Similarly, the use of the more sensitive TAC molecular diagnostic could increase the apparent rate of microbiologically confirmed VAP, it is reassuring that TAC was used marginally more frequently in the non-COVID patients and this also suggests the difference seen is due to biological rather than technical reasons.

Reports of rates of VAP amongst ventilated patients with COVID-19 vary, with rates of 40–86% reported [38–40] and our reported rate of 49% is in keeping with reports from other centres. Although some reports, not focussed specifically on VAP, indicate lower rates of 10% [8], it is unclear how many of the ICU patients in that cohort were ventilated for at least 48 h. The rates of VAP between centres managing COVID-19 are likely to vary depending on the clinical characteristics of the patients managed, differential ICU admissions policies and clinical factors such as use of immunosuppressive therapies. Although we managed to maintain one to one nursing ratios throughout the first wave of COVID-19, it is possible that the increased numbers of nurses with only brief training in critical care led to increased rates of VAP. However, the continued high compliance with the ventilator care bundle which includes key nursing interventions such as oral hygiene and head of bed elevation argues against this being a major factor. We acknowledge the sample size and single centre limitations with our observations and suggest larger studies from distinct geographic locations may help fully understand the risk of developing secondary bacterial infections in patients with severe COVID-19.

## Conclusion

COVID-19 makes people more susceptible to developing VAP, partly but not entirely due to the increased duration of ventilation. The change in lung microbiome and causes of secondary infection are similar to those seen in critically ill patients ventilated for other reasons. Careful sampling of the respiratory tract whilst minimising contamination from the proximal tract, in combination with sensitive diagnostic testing to reduce the risk of false negative cultures will aid antimicrobial optimisation in patients with COVID-19.

## Supplementary information

**Additional file 1. **Supplemental methods and results.

## Data Availability

Raw sequencing data has been deposited at https://www.ncbi.nlm.nih.gov/bioproject/PRJNA642012.
