# Correction to: Ventilator-associated pneumonia in critically ill patients with COVID-19

**DOI:** 10.1186/s13054-021-03560-2

**Published:** 2021-04-06

**Authors:** Mailis Maes, Ellen Higginson, Joana Pereira-Dias, Martin D. Curran, Surendra Parmar, Fahad Khokhar, Delphine Cuchet-Lourenço, Janine Lux, Sapna Sharma-Hajela, Benjamin Ravenhill, Islam Hamed, Laura Heales, Razeen Mahroof, Amelia Soderholm, Sally Forrest, Sushmita Sridhar, Nicholas M. Brown, Stephen Baker, Vilas Navapurkar, Gordon Dougan, Josefn Bartholdson Scott, Andrew Conway Morris

**Affiliations:** 1 Department of Medicine, Cambridge Institute of Therapeutic Immunology and Infectious Disease (CITIID), University of Cambridge, Cambridge, UK; 2 Clinical Microbiology and Public Health Laboratory, Public Health England, Addenbrooke’s Hospital, Cambridge, UK; 3 Division of Anaesthesia, Department of Medicine, University of Cambridge, Level 4, Addenbrooke’s Hospital, Hills Road, Cambridge, CB2 0QQ UK; 4 John Farman ICU, Addenbrookes Hospital, Cambridge, UK; 5 Wellcome Sanger Institute, Hinxton, UK

## Correction to: Crit Care (2021) 25:25 10.1186/s13054-021-03460-5

Following publication of the original article [1], the authors reported an error in one of the contributing author names. The revised author name is indicated hereafter and the changes have been highlighted in **bold typeface**.

The incorrect author name reads:

Amelia Solderholm.

The correct author name should read:

Amelia **Soderholm.**

All the changes requested are implemented in this correction and the original article [1] has been corrected.
