# Supplementary material for: Ventilator-associated pneumonia in critically ill patients with COVID-19
Source: Crit Care. 2021 Jan 11;25:25. doi: 10.1186/s13054-021-03460-5 (PMC7797892; doi:10.1186/s13054-021-03460-5)
Supplement: Supplementary file 1 — Additional file 1. Supplemental methods and results. [file 13054_2021_3460_MOESM1_ESM.pdf]

Ventilator-associated pneumonia in critically ill patients with COVID-19 -Supplemental material

Supplemental methods

RNA/DNA extraction and SARS-CoV-2 qPCR

500µl of BAL was subjected to RNA/DNA extraction following an existing method [E1] . Viscous samples were first treated with 10% v/v mucolysin, before 500µl lysis buffer (25mM Tris-HCL+ 4M Guanidine thiocyanate with 0.5% b-mercaptoethanol) and glass beads were added to each sample. Tubes were vortexed, and 100% analytical grade ethanol was added to a final concentration of 50%. After a 10 min incubation, 860µl of lysis buffer (containing MS2 bacteriophage as an internal extraction and amplification control) was added. This was then run over an RNA spin column as previously described [E2]. After validating the automated NUCLISENS easyMAG platform (Biomerieux, Marcy L'Etoile, France) for safe extraction sample processing was switched to this automated platform still using 500µl input sample and inclusion of the MS2 internal control SARS-CoV-2 specific real-time RT-PCR was performed and interpreted as previously described [E2]

TaqMan multi-pathogen array

Custom designed TaqMan Array Cards (TAC; Thermo Fisher Scientific) targeting 52 different common respiratory pathogens, were used to test for secondary infections as previously described [E1]. Fifty microlitres of extracted nucleic acid was used in a 200 µl final reaction volume with TaqMan™ Fast Virus 1-step Master Mix (Thermo Fisher Scientific), and cards were run on the QuantStudio 7 Flex platform (Thermo Fisher Scientific) as per the manufacturer's instructions. Detection of a clear exponential amplification curve with a Cycles to Threshold (CT) value ≤ 32 for any single gene target was reported as a positive result for the relevant pathogen. We have previously demonstrated that CT value of ≤ 32 corresponded to growth ≥ 10^4/CFU/ml, hence the use of this threshold to define VAP [E1].

16S Nanopore sequencing

Extracted nucleic acids were concentrated using AMPure XP beads (Beckman Coulter) and 16S DNA libraries prepared using the 16S Barcoding Kit SQK-16S024 (Oxford Nanopore Technologies) as per the manufacturer's instructions. Final DNA libraries were loaded onto FLO-MIN106D R9.4.1 flow cells and sequencing was performed on a GridION Mk1 for ~36 hours with high accuracy basecalling enabled. The resulting fastq files were de-multiplexed with guppy_barcoder v3.6.0 using the --require_barcodes_both_ends and --trim_barcodes flags. Porechop v0.2.4 (https://github.com/rrwick/Porechop) was used to trim adapter and barcode sequences and Nanofilt v2.6.0 (De Coster, et. al., 2018) was used to filter the reads by length, 1,400 – 1,600 bps and a quality score of 10. Reads were classified against the Silva 132 99% Operational Taxonomic Units (OTUs) 16S database using Kraken2[E3]. Microbial diversity analyses were carried out in R using packages vegan [E4] and metacoder [E5].

Supplemental tables and figures

Primary reason for admission	number
Abdominal surgical emergency	28
Pneumonia/pneumonitis	23
Liver transplantation	19
Neurological emergency	13
Hepatic failure	12
Post-Cardiac arrest	12
Trauma	8
Infection -other source	8
Toxin ingestion	6
Vascular surgical emergency	6
Cardiogenic shock/pulmonary oedema	3
Other pathologies	4
Urinary tract infection/obstruction	2

Table S1: primary admission categories for 144 patients admitted without COVID-19

Parameter	Covid (39)	Non-COVID (19)
Median duration of ventilation prior to VAP (IQR)	9 (6-13)	5 (4-9)
(when diagnosed by bronchoscopy)	8 (5-13)	5 (4-7)
(when diagnosed by ETA)	9 (6-14)	8 (3-12)
% 'early' VAP (<96 hours of ventilation)	13%	33%
Median P/F ratio at time of diagnosis (IQR)	17 (13-21)	23 (17-25)
Antibiotics in 72 hrs prior to diagnosis	83%	89%
Antibiotics on day of sampling	80%	89%
% diagnosed by bronchoscopy	20 (51%*)	10 (53%**)

*4 cases not assessed by TAC due to lack of availability of laboratory capacity
** 1 case not assessed by TAC due to lack of availability of laboratory capacity

Table S2: details of ventilator-associated pneumonias detected in patients with and without COVID-19

Parameter	COVID (n=64)	Non-COVID (n=48)	P value (z-test for proportions)
% of all patients	79%	34%	<0.0001
% microbiologically confirmed VAP	39 (61%)	19 (40%)	0.02
% investigated which met clinical and radiological VAP criteria (figure 1)	59 (92%)	39 (81)%	0.08
Median duration of ventilation prior to investigation for VAP (IQR)	7 (5-12)	6 (4-9)	0.06
Median P/F ratio at time of investigation (IQR)	18 (12-21)	23 (18-30)	0.0005
Antibiotics in 7 days prior to investigation	54 (84%)	45 (94%)	0.12
Antibiotics in 72 hrs prior to investigation	47 (74%)	40 (83%)	0.21
% investigated by bronchoscopy	30 (47%*)	23 (48%**)	0.91
Incidence of microbiologically confirmed VAP by BAL	20 (25%)	10 (7%)	0.0002
% of microbiologically confirmed VAP with new consolidation on CXR	26 (67%)	19 (100%)	0.004
% of microbiologically confirmed VAP with deteriorating diffuse shadowing on CXR	13 (33%)	0 (0%)	0.004

Table S3: Details of patients with and without COVID-19 investigated for suspected VAP
*4 cases not assessed by TAC due to lack of availability of laboratory capacity
** 1 case not assessed by TAC due to lack of availability of laboratory capacity

	**COVID**	**Non-COVID**
Ceftriaxone	0	1
Ciprofloxacin	3	8
Co-amoxiclav	13	10
Cotrimoxazole	0	1
Clarithromycin	7	1
Meropenem	10	11
Metronidazole	1	3
Piperacillin/tazobactam	32	20
Vancomycin	5	5
Antifungal (azole or echinocandin)	1	4

Table S4: Antimicrobial exposure in the 7 days prior to investigation for VAP

**Parameter**	**COVID-19 (n=81)**	**Non-COVID-19 (n=144)**	**Univariable P value**	**Adjusted P value in multivariable Cox model**
COVID			**0.015**	0.045
Median Age (IQR)	62 (50-70)	62 (49-72)	0.986	-
Sex (% female)	31%	40%	**0.05**	0.123
Hypertension	33%	33%	0.349	-
Diabetes	22%	24%	0.927	-
Obesity	37%	24%	0.425	-
Chronic Kidney Disease	12%	9%	0.544	-
Chronic lung disease	19%	24%	0.175	-
Immunocompromised	15%	25%	**0.038**	0.076
Corticosteroid use in ICU	16%	16%	0.599	-
Median APACHE II (IQR)	15 (11-19)	16 (12-20)	0.06	-
% with ARDS on ICU admission	63 (78%)	22 (15%)	0.625	-
% ventilated prone	40 (49%)	1 (0.7%)	0.18	-
Median P/F ratio in 24 hrs following admission	18 (13-28)	34 (24-37)	0.301	-
Antibiotics in 24 hrs following admission	76 (94%)	126 (88%)	0.09	-

Table S5: Variables evaluated for association with development of VAP in a Cox proportional hazards model with censoring for death and extubation. Variables were entered in a univariable fashion into the Cox model, and those with a p value of </=0.05 were included in the final model, the adjusted p values from this final model shown in the right hand column.

Patient	Sample	SARS-CoV-2*	COVID-19**	Organism with Ct ≤32	Cultured at $\geq 10^4$ CFU/ml	VAP
Patient 1	1	POS	YES	S. aureus	No significant growth	YES
	2	POS	YES	S. maltophilia	No significant growth	YES
Patient 2	1	POS	YES	H. influenzae	No significant growth	YES
Patient 3	1	POS	YES	K. pneumoniae, S. marcescens, E. coli S. epidermidis	No significant growth	YES
Patient 4	1	POS	YES		No significant growth	NO
	2	POS	YES	P. aeruginosa	P. aeruginosa	YES
Patient 5	1	POS	YES	HSV, S. epidermidis, C. albicans	C. albicans	NO
Patient 6	1	POS	YES	S. aureus, S. epidermidis, Streptococcus spp	S. aureus	YES
	2	POS	YES	S. aureus, E. faecium, S. marcescens	S. aureus	YES
	3	POS	YES	S. aureus, E. faecium, HSV	S. aureus, S. maltophilia	YES
Patient 7	1	POS	YES	E. faecium, S. epidermidis	Mixed upper resp. tract flora	NO
Patient 8	1	POS	YES	E. faecium, C. albicans	C. albicans	NO
Patient 9	1	POS	YES	HSV, S. marcescens,	S. marcescens	YES
	2	POS	YES	E. faecium, S. marcescens	No significant growth	YES
Patient 10	1	POS	YES	K. pneumoniae	No significant growth	YES
Patient 11	1	POS	YES		No significant growth	NO
Patient 12	1	POS	YES	A. fumigatus, S maltophilia	S. maltophilia	YES
Patient 13	1	POS	YES		No significant growth	NO
Patient 14	1	POS	YES		No significant growth	NO
	2	NEG	YES	K. pneumoniae	K. pneumoniae	YES
	3	NEG	YES	K. pneumoniae	K. pneumoniae	YES
Patient 15	1	NEG	NO	C. albicans	C.albicans	NO
Patient 16	1	NEG	NO		E. coli	YES
Patient 17	1	NEG	NO	S. marcescens	Serratia marcescens	YES
Patient 18	1	NEG	YES	Streptococcus spp, E. Proteus , K. pneumoniae	No significant growth	YES
Patient 19	1	NEG	YES	E. faecium, E. coli	E. coli	YES
Patient 20	1	NEG	YES	Enterobacteriaceae	No significant growth	YES
Patient 21	1	NEG	NO	Streptococcus spp, H. influenzae	No significant growth	YES
Patient 22	1	NEG	NO	H. influenzae, Streptococcus spp, S. aureus	No significant growth	YES
Patient 23	1	NEG	NO		No significant growth	NO
	2	NEG	NO		No significant growth	NO
Patient 24	1	NEG	NO	HSV	No significant growth	NO
	2	NEG	NO	HSV	No significant growth	NO
Patient 25	1	NEG	YES		No significant growth	NO

Table S4: details of the organisms detected by TaqMan array and culture in the patients included in the 16s sequencing sub-study. * positive for SARS-CoV-2 by PCR at time of sampling, ** admitted to ICU with COVID-19 confirmed by SARS-CoV-2 PCR.

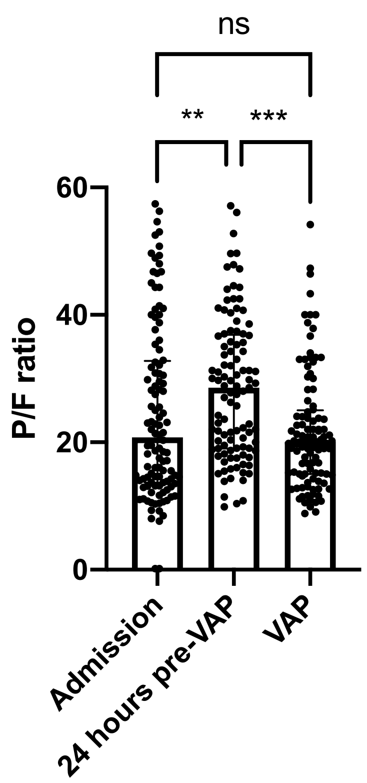

Figure S1: P/F ratio in patients investigated for suspected VAP at ICU admission, 24 hours prior to investigation for VAP and on day of investigation for VAP. P <0.0001 by Kruskal-Wallis, ** P=0.0019, *** P<0.0001, NS p=0.99 by Dunn's post-hoc test.

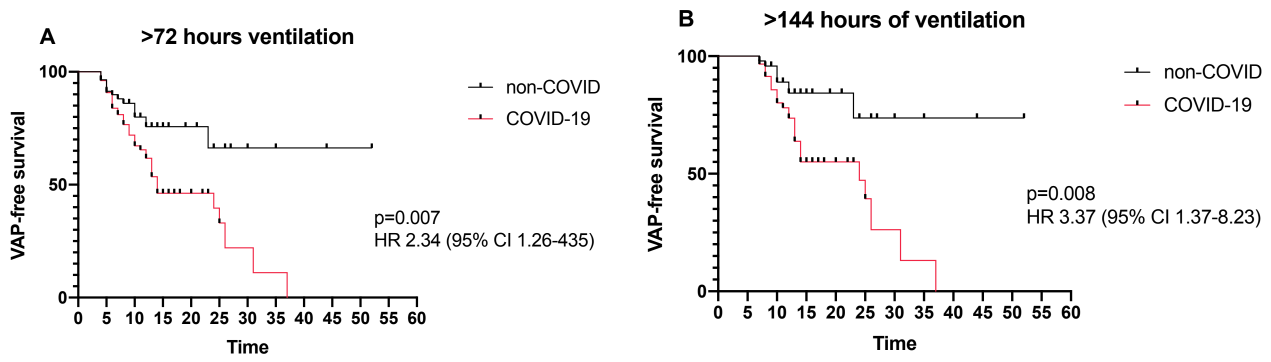

Figure S2: Survival curves for subsets of patients ventilated for at least 72 hours (A) and at least 144 hours (B). HR, hazard ratio. P values by Cox proportional hazards model

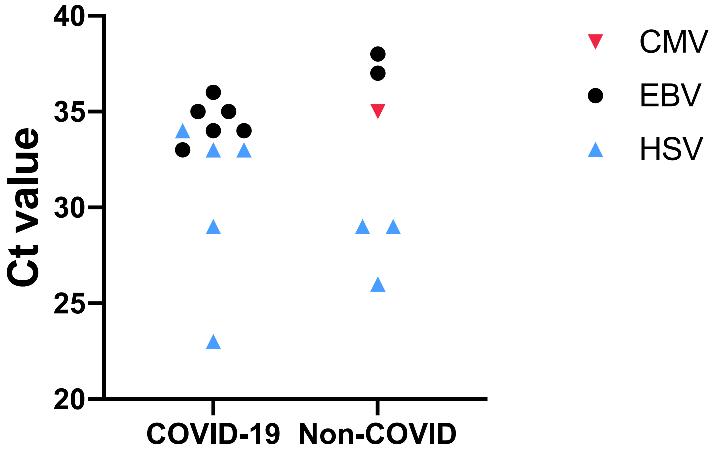

Figure S3: Cycles to crossing threshold (Ct) for herpesvirade detected on the TAC in bronchoalveolar lavage from patients with and without COVID-19. CMV -cytomegalovirus, EBV-Epstein-Barr virus, HSV-herpes-simplex virus. From 15 patients with positive detection (2 patients had detection of both HSV and EBV).

Supplemental references

E1 Navapurkar V, Bartholdson-Scott J, Maes M, et al. Development and implementation of a customised rapid syndromic diagnostic test for severe pneumonia. medRxiv. June 2020:1-21. doi:10.1101/2020.06.02.20118489.

E2 Rivett L, Sridhar S, Sparkes D, et al. Screening of healthcare workers for SARS-CoV-2 highlights the role of asymptomatic carriage in COVID-19 transmission. Elife. 2020;9. doi:10.7554/eLife.58728.

E3 Wood DE, Lu J, Ben Langmead. Improved metagenomic analysis with Kraken 2.Geneome Biol. 2019:1-13.

E4 Oksanen, J. (2013). Vegan: ecological diversity. R Project.

E5 Foster ZSL, Sharpton TJ, Grünwald NJ. Metacoder: An R package for visualization and manipulation of community taxonomic diversity data. Poisot T, ed. PLoS Comput Biol. 2017;13(2):e1005404-e1005415.
